# The Lysine Deprotonation Mechanism in a Ubiquitin Conjugating Enzyme

**DOI:** 10.1021/acs.jpcb.5c01486

**Published:** 2025-05-12

**Authors:** Alexis J. Wathan, Nicole M. Deschene, Joseph M. Litz, Isaiah Sumner

**Affiliations:** † Department of Science and Mathematics, Rochester Institute of Technology/NTID, Rochester, New York 14623, United States; ‡ Department of Chemistry and Biochemistry, James Madison University, Harrisonburg, Virginia 22807, United States

## Abstract

Ubiquitination is a biochemical reaction in which a small protein, ubiquitin (Ub), is covalently linked to a lysine on a target protein. This type of post-translational modification can signal for protein degradation, DNA repair, or inflammation response. Ubiquitination is catalyzed by three families of enzymes: ubiquitin activating enzymes (E1), ubiquitin conjugating enzymes (E2), and ubiquitin ligases (E3). In this study, we focus on the chemical mechanism used by the E2 enzyme, Ubc13, which forms polyubiquitin chains by linking a substrate Ub to Lys63 on a target ubiquitin (Ub*). Initially, Ubc13 is covalently linked to the substrate Ub. Next, Lys63 in the Ub* is deprotonated, becomes an active nucleophile, and attacks the thioester bond in the Ubc13∼Ub conjugate. The deprotonation mechanism is not well understood. There are two, conserved nearby residues that may act as conjugate bases (Asp119 on Ubc13 and Glu64 on Ub*.) It is also hypothesized that the active site environment suppresses the lysine’s p*K*_a_, favoring deprotonated lysine. We test these hypotheses by simulating both WT and mutant Ubc13 with constant pH molecular dynamics (CpHMD), which allows titratable residues to change their protonation states. In our simulations, we have five titratable residues, including Lys63, and we use these simulations to monitor the protonation states and to generate titration curves of lysine 63. We found that the p*K*_a_ of Lys63 is highly dependent on its distance from the active site. Also, mutating Asp119 or Glu64 to Ala has little effect on the lysine p*K*_a_, indicating that neither residue acts as a generalized base. Finally, we note that mutating a structural residue (Asn79 to Ala) increases the lysine p*K*_a_, suggesting that alterations to the active site hydrogen bonding network can affect nucleophile activation.

## Graphical abstract

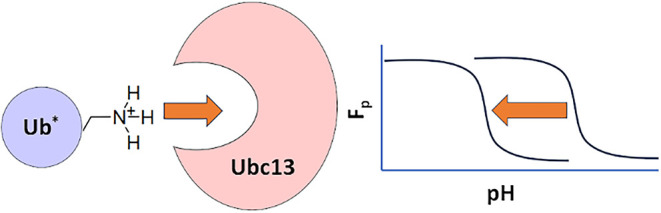

## I Introduction

Ubiquitination is a biochemical reaction in which the small regulatory protein ubiquitin (Ub) is attached to a target protein.^1–3^ This process either attaches a single Ub (monoubiquitination), or a chain of ubiquitins (polyubiquitination). In both cases, the C-terminal glycine of Ub is linked to an amino acid on the target. If the target is also Ub, as happens in polyubiquitination, an isopeptide bond forms between the C-terminal glycine of the transferring Ub and a lysine on the target Ub*. (In this paper, we denote the target ubiquitin with an asterisk, Ub*). Polyubiquitination can be complex because Ub* has seven different lysines–K6, K11, K27, K29, K33, K48 and K63–so many types of linkages are possible. Because of the manifold ways that ubiquitination can occur, it is able to regulate many different biochemical pathways. For example, K48-linked polyubiquitin chains signal for protein degradation, whereas K63-linked polyubiquitin chains signal for several processes including for DNA repair and inflammation response.^4–11^

Ubiquitination is sequentially catalyzed by three families of enzymes: ubiquitin activating enzymes (E1), ubiquitin conjugating enzymes (E2), and ubiquitin ligases (E3). In this work, we are focused on the second step of the ubiquitination cascade catalyzed by the E2 enzyme. Specifically, we probe the mechanism used by the E2, Ubc13,^12^ which catalyzes the formation of K63-linked polyubiquitin chains. At the beginning of this step, the C-terminus of Ub is linked to the side chain of C87 in Ubc13 via a thioester bond. Ubc13 then catalyzes the breaking of the thioester bond and the formation of an isopeptide peptide bond between the C-terminus of Ub and the side chain of K63 of the target Ub*. The chemistry is outlined in Scheme 1. This step is aided by a RING-E3 ligase, which improves the overall catalytic efficiency, possibly by placing tension on the thioester bond, lowering the energy required for it to break.^13,14^

**Scheme 1:**
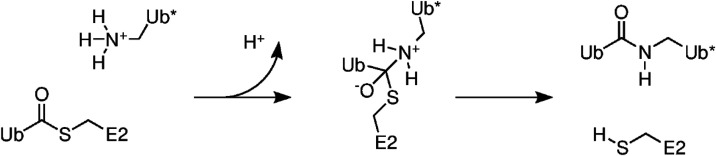
Simplified Reaction Scheme for Ubc13-Catalyzed Ubiquitination^a^

a In the first step, the amine nucleophile is activated (deprotonated) and attacks the carbonyl carbon in the thioester bond, forming a tetrahedral, zwitterionic intermediate. The intermediate collapses, and an isopeptide bond is formed between Ub and its target, Ub*.

Ubc13 is thought to catalyze Ub transfer by stabilizing the formation of zwitterionic intermediate. The side chain of a highly conserved asparagine (N79 in Ubc13) is hypothesized to stabilize the negative charge.^12,15–19^ A complementary hypothesis is that N79 is the linchpin in a network of hydrogen bonds that preorganize the active site.^14,20–22^ However, the first step in the reaction—the activation of the amine nucleophile—is less well-understood.

There are a few proposed mechanisms for this step. One hypothesis is that the lysine is actively deprotonated by a generalized base.^4,13,19,23–27^ In Ubc13, D119 is a possibility since it is near the active site. E64 in the target Ub* is another candidate, since it is adjacent to the nucleophile, K63. A second hypothesis is that the environment of the active site suppresses the p*K*_a_ of K63, making it energetically more favorable to deprotonate.^4,17,27–30^ In this work, we probed both conjectures by using constant pH molecular dynamics (CpHMD)^31^ simulations to allow for five key residues (including K63) to change their protonation states. We conducted computational experiments to determine the p*K*_a_ of the substrate lysine in WT and mutant enzymes. We also measured the lysine deprotonation probability as it enters the enzyme binding pocket. Our simulations support the p*K*_a_ suppression hypothesis.

## II Methods

### II.I Structural Preparation

All initial coordinates were taken from a structure deposited in the protein databank (PDB code 5AIT).^32^ Chains A and E–G were deleted and a crystal packing partner representing the target Ub (Ub*) was added. The final structures contained an E2­(Ubc13)∼Ub conjugate, a UeV (UbeV2), and a target Ub (Ub*) (see Figure 1). In total, four different initial structures were constructed: WT, Ubc13 mutants-D119A and N79A, and the Ub* mutant–E64A. Mutants were created by deleting the relevant side chain coordinates and relabeling the backbone atoms as the substituted amino acid. The LEaP module in AmberTools14 ^33^ was used to add missing hydrogen and heavy atoms to the systems, to solvate them in a truncated octahedral box of TIP3P waters with a 10 Å buffer, and to neutralize them with 16 Cl^–^ and 15 or 16 Na^+^ ions.

**Figure 1:**
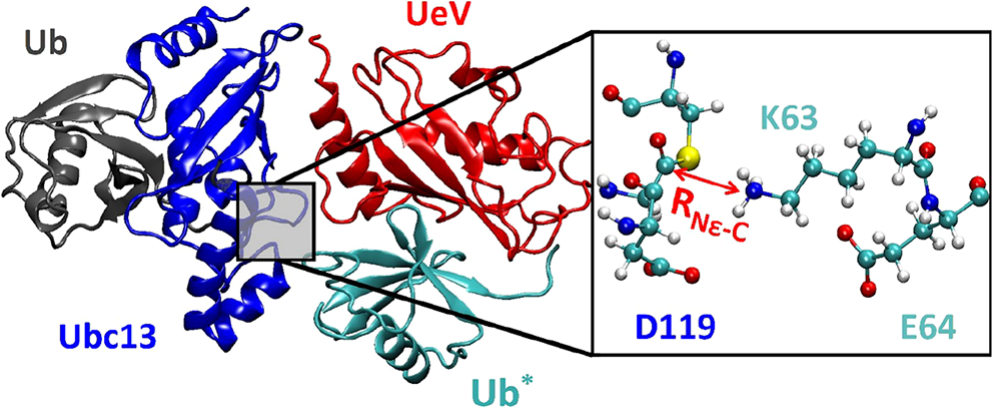
Model system is shown on the left with each protein colored differently. The E2, Ubc13, is dark blue. Ub is gray. The ubiquitin enzyme variant, UbeV2, is red and the target ubiquitin, Ub*, is cyan. The right panel is a zoomed-in look at the active site. The putative general bases, D119 on Ubc13 and E64 on Ub*, are highlighted. Additionally, the distance between N_ε_ on K63 in Ub* and the thioester carbon in Ubc13∼Ub, *R*_N_ε_–C_, is indicated with a red arrow.

The systems were simulated using the Amberff10 force-field. ff10 was chosen to ensure compatibility with the constant pH MD algorithm.^31^ The thioester bond between the Ubc13 and Ub was represented with custom force-field parameters developed in refs 14,21,22 and available in the Supporting information (SI). Briefly, we used RESP charges,^34^ parameters taken from GAFF,^35^ and a custom improper torsion benchmarked against DFT.^14^

To understand the how the environment around the substrate lysine changes as it enters the active site, we controlled the distance between N_ε_ on K63 in Ub* and the thioester carbon in Ubc13∼Ub (*R*_N_ε_–C_) for some WT simulations. See Figure 1 for an illustration of this coordinate. We initially used 5 different windows: *R*_N_ε_–C_ = 0–3, 2–4, 3–5, 5–7, and 7–9 Å. Within each window, the distance was unrestrained, and for distances 2 Å less than the window and 2 Å greater than the window the restraining force was harmonic with a force constant of 16 kcal mol^–1^ Å^–2^. Beyond that, the restoring force was linear. Due to a gap in coverage, we used a sixth window where *R*_N_ε_–C_ was constrained to 3.5 Å with a harmonic force constant of 15 kcal mol^–1^ Å^–2^. Figure S2 in the SI shows the distribution of R_Nε–C_ across all simulation windows.

After their construction, each structure (WT, mutant, unrestrained and restrained *R*_N_ε_–C_) was optimized for 5000 steps with harmonic restraints of 10 kcal mol^–1^ Å^–2^ placed on the protein backbone. Next, the backbone restraints were removed, and each system was heated from 10 to 300 K over 300 ps; the temperature was then held constant for an additional 100 ps. Finally, each system was equilibrated using NPT dynamics at 1 atm and 300 K for 4 ns.

### II.II Constant pH Molecular Dynamics

To examine the effect of environment on the K63 protonation state, we ran explicit solvent constant pH Molecular Dynamics (CpHMD)^31^ at pH 7.0 for the WT system with *R*_N_ε_–C_ restrained. We ran 4, independent 100 ns CpHMD simulations for each distance window, for a total of 2.4 μs of simulation. (Each was prepared with independent optimizations, heating and NPT equilibration steps.) To reduce the computational expense, only K94, D118, D119 in Ubc13 and K63 and E64 in Ub* were protonatable. Protonation changes were attempted every 100 steps (200 fs), and the explicit solvent relaxation time was 200 fs. Backbone RMSDs are shown in the SI (Figures S3–S8).

### II.III pH-REMD

To quantify the p*K*_a_ of the substrate lysine, we ran explicit solvent pH-Replica Exchange Molecular Dynamics (pH-REMD) for mutant (D119A, E64A, and N79A) and WT enzymes.^31^ Unless the residue was mutated to Ala, K94, D118, D119 in Ubc13 and K63 and E64 in Ub* were titratable. We ran pH replicas from pH 6.0 to 11.5, with a replica at each 0.5 pH units. Each simulation was 120 ns long. Exchanges between replicas were attempted every 100 steps (200 fs), protonation changes were attempted every 200 fs, and the explicit solvent relaxation time was 200 fs. pH-REMD was run for the WT and N79A enzymes–unrestrained and restrained to the *R*_N_ε_–C_ = 0–3 and 3.5 Å windows. For the three mutants, we only ran restrained simulations in the 3.5 Å window. Figure S9 in the SI shows the distribution of *R*_N_ε_–C_ for each pH-REMD simulation.

All MD was run using the GPU-accelerated pmemd module of Amber14,^33^ Amber20,^36^ or Amber24.^37^ Analyses were carried out using the CPPTRAJ program in AmberTools^38^ and using Gnuplot5.0.^39^ Input for all simulations are available in the SI. Trajectory files are only available upon request due to their size (>1TB).

## III Results and Discussion

### III.I The Protonation State of K63 Depends on Its Distance from the Active Site

To ascertain a dependence between K63’s protonation state and its distance from the active site, we ran CpHMD simulations with the distance between the N_ε_ on K63 in Ub* and the thioester carbon in Ubc13∼Ub (*R*_N_ε_–C_) restrained to stay within certain distance windows. We then monitored the fraction of the simulation that K63 is protonated within each window. The results are pictured in Figure 2. K63 remains mostly protonated until it is within 3 Å of the thioester carbon, at which point it is mostly deprotonated—only 7.6% of the trajectory has a protonated lysine. The inflection point occurs at around 3.5 Å. In the 3.5 Å window, K63 is protonated on average for 63.8 ± 23% of the trajectory. Furthermore, the putative bases remain largely deprotonated independent of *R*_N_ε_–C_. Across all distance windows, D119 is deprotonated 98.0% of the time and E64 is deprotonated 94.2% of the time. One possible exception is E64 in the 3.5 Å window. The large standard deviation arises from one of the four simulations in which E64 is deprotonated for only 42.0% of the trajectory. In this same simulation, the K63 is protonated for 86.3% of the trajectory, which is on the high end of the distribution.

**Figure 2:**
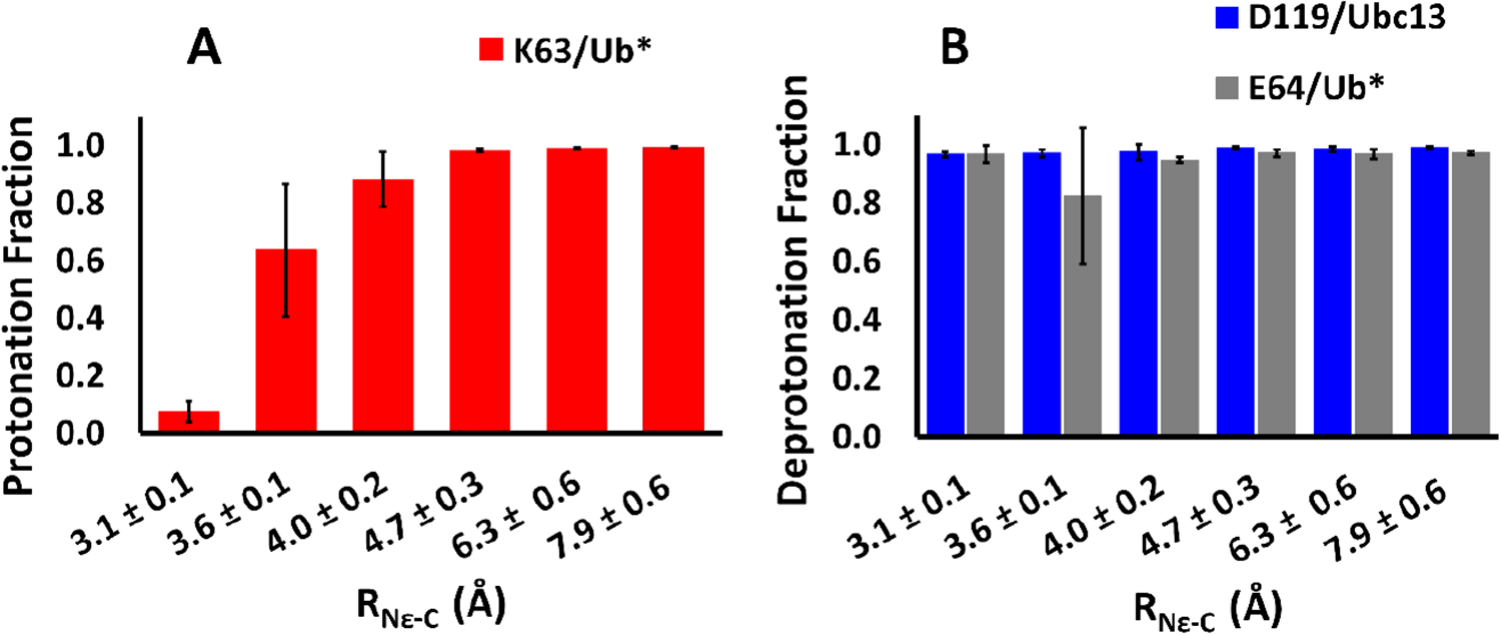
(A) Fraction of the simulation that the substrate lysine, K63, is protonated decreases as it enters the active site, i.e., as *R*_N_ε_–C_ decreases. (B) The putative general bases, D119 and E64, are *de*protonated at all values of *R*_N_ε_–C_. The error bars are the standard deviations across the 4, independent simulations conducted in each distance window. The *x*-axis is labeled by the average *R*_N_ε_–C_ in each window along with the standard deviation. K63 is more likely to lose its proton at short distances, whereas D119 and E64 show no dependence on distance.

To further quantify the relationship between *R*_N_ε_–C_ and K63’s protonation state, we calculated the point-biserial correlation coefficient, *r*_pb_, which measures the correlation between a binary variable (protonation) and a continuous variable (distance).^40^ *r*_pb_ is defined in eq 1

1 $$r_{pb}=\frac{{\left\langle R_{N_{\varepsilon }\text{−}C}\right\rangle }_{d}-{\left\langle R_{N_{\varepsilon }\text{−}C}\right\rangle }_{p}}{\sigma }\sqrt{\frac{N_{d}N_{p}}{N^{2}}}$$

where ⟨*R*_N_ε_–C_⟩_d_ is the average *R*_N_ε_–C_ distance when K63 is *de*protonated, ⟨*R*_N_ε_–C_⟩p is the average *R*_N_ε_–C_ distance when K63 is protonated, σ is the *R*_N_ε_–C_ standard deviation, *N*_d_ is the number of frames with K63 *de*protonated, *N*_p_ is the number of frames with K63 protonated, and *N* = *N*_p_ + *N*_d_ the total number frames. This data is presented in Figure 3.

**Figure 3:**
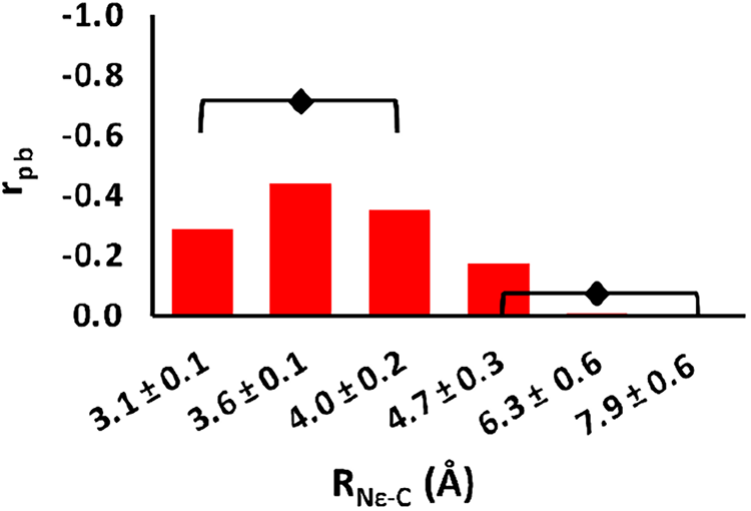
Bars indicate the value of the point-biserial correlation coefficient, *r*_pb_, in each of the K63-thioester (*R*_N_ε_–C_) distance windows. All four simulations in each window were used to calculate *r*_pb_. There is more correlation between distance and K63 protonation at smaller distance (≤4.0 Å) than at larger distances. The black diamond indicates the correlation taking all the bracketed distances windows into account. The correlation for the closest 3 distances is quite high, −0.712, especially compared to the longer distances, −0.073. The negative correlation means that as the distance decreases, the lysine is deprotonated.

Figure 3 shows that there is a high correlation between the protonation state of K63 and its distance from the thioester carbon in the Ubc13∼Ub conjugate (*R*_N_ε_–C_). The correlation is low for individual distance windows (red bars in Figure 3), but when several windows are considered, the correlation increases (black diamonds). When the correlation is calculated for 0–3, 3.5, 2–4 Å windows simultaneously, the correlation is strong, −0.712—the negative correlation means that as the distance decreases, the lysine is deprotonated. At longer distances, there is no correlation. This demonstrates that K63 tends to lose its proton as it moves closer to the thioester.

### III.II The p*K*_a_ of K63 is Distance Dependent

Next, we quantified the p*K*_a_ of K63 as a function of distance using pH-REMD simulations. These simulations allow for the construction of a titration curve, which can be fit to the Hill equation

2 $$F_{p}=\frac{1}{1+10^{n\left(pH-pK_{a}\right)}}$$

where *F*_p_ is the fraction protonated and *n*–the Hill coefficient–and p*K*_a_ are fitting parameters. A Hill coefficient less than one can be used to infer cooperativity between titratable residues. We used GnuPlot5.0 ^39^ to fit the equation and the results of these simulations are shown in Figure 4.

**Figure 4:**
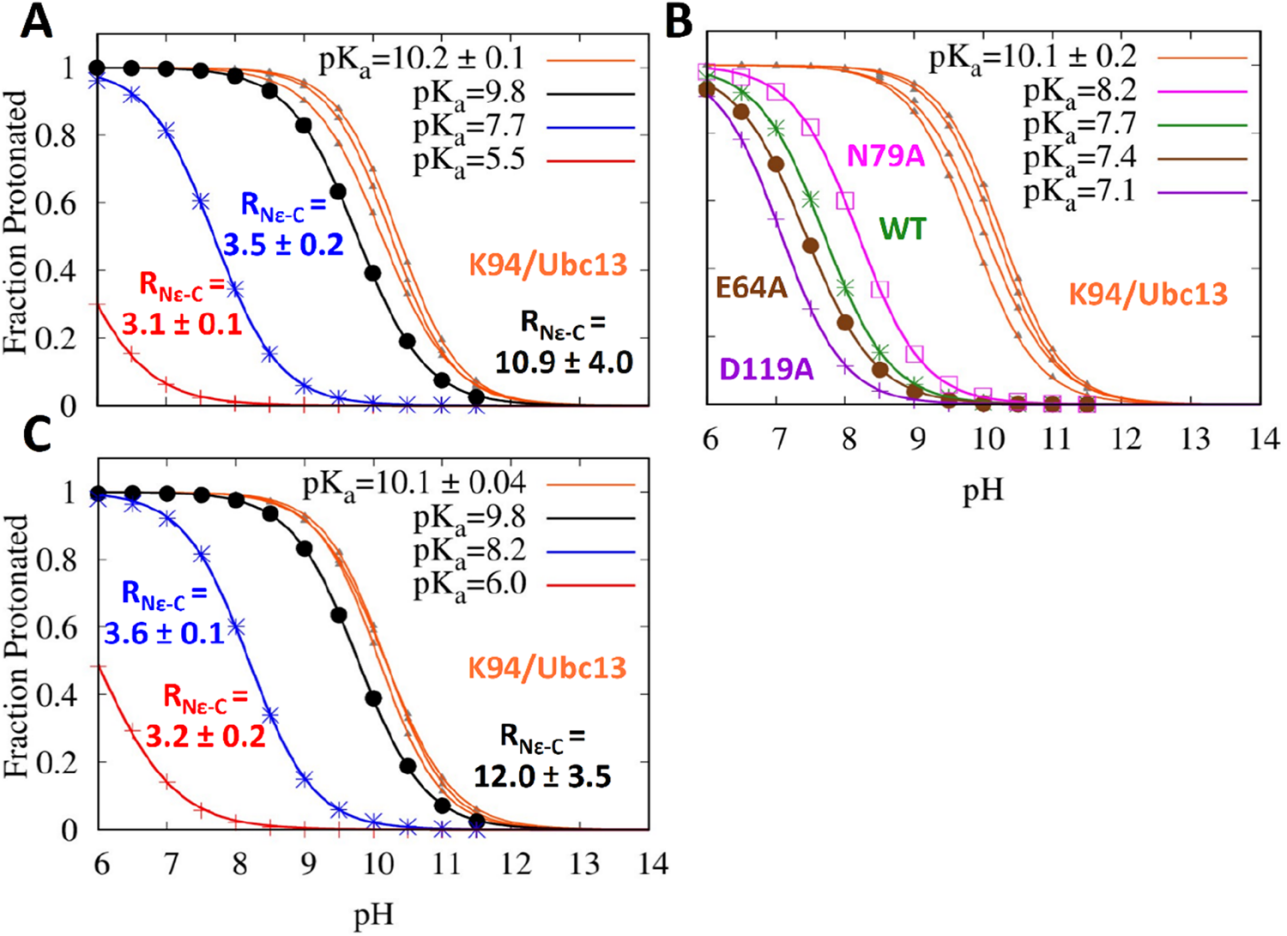
(A) p*K*_a_ of K63 decreases as the distance from the active site decreases, (B) point mutations have a small effect on the p*K*_a_, and (C) the p*K*_a_ of K63 in the N79A mutant active site is elevated compared to the WT by ∼0.5 p*K*_a_ units. In (A) (WT) and (C) (N79A), the blue and red curves were calculated at the 3.5 and 0–3 Å windows; the black curve was unrestrained. These curves are labeled by the average and standard deviation of *R*_N_ε_–C_ calculated in three of the pH replica windows. In (B), all calculations were performed in the 3.5 Å window. The magenta, purple, and brown curves represent N79A/Ubc13, D119A/Ubc13, and E64A/Ub* point mutations, and the green curve is the WT enzyme. In (A–C), the orange titration curves are for K94 in Ubc13 and the average p*K*_a_ of K94 plus the standard deviation is shown. K94 acts as a control, since its p*K*_a_ should not change.

Because the protonation state of K63 begins to change in the 3.5 Å window, we calculated p*K*_a_s only in the 0–3, 3.5 Å windows, as well as a simulation in which the K63 was unrestrained. We also monitored the p*K*_a_ of K94 in Ubc13. Because K94 is always unrestrained, it should act as a control. (Across all 8 pH-REMD simulations, the p*K*_a_ of K94 is 10.1 with a standard deviation of 0.1.) As is clear from Figure 4A, the p*K*_a_ is suppressed as the substrate lysine enters the active site. The p*K*_a_ of the unrestrained simulation is 9.8, which is near the baseline p*K*_a_ of lysine in solution, 10.5. The p*K*_a_ is greatly reduced when K63 is placed in the active site, where the p*K*_a_ becomes 7.7 and 5.5. For all these simulations, the p*K*_a_ of K94 remains unchanged at 10.2 ± 0.1. The Hill coefficient was 0.881 for the unrestrained simulation, 0.911 for 3.5 Å, and 0.794 for 0–3 Å. For K94, the Hill coefficient was 0.94 ± 0.06. A Hill coefficient of 1 indicates no cooperativity between titratable residues. Therefore, we can conclude that a generalized base does not deprotonate K63. We note that the pH-REMD simulations only had replicas from pH 6–11.5, so a p*K*_a_ of 5.5 is extrapolated in the 0–3 Å window, adding a degree of uncertainty for both the Hill coefficient and the p*K*_a_ of this simulation. This presumption is supported by the high degree of correlation between the fitted Hill coefficient and the p*K*_a_ in this window; the correlation factor is −0.892. By contrast, the correlation between *n* and p*K*_a_ for all the other fits range from −0.024 to 0.01, suggesting that the low Hill coefficient for the 0–3 Å window may be a result of poor coverage in the region where pH ≈ p*K*_a_.

We also measured the p*K*_a_ of K63 in mutant enzymes. We mutated the putative bases, D119 in Ubc13 and E64 in Ub, to alanine. We also mutated N79 in Ubc13 to alanine. N79 is known to play an important role in the catalytic function of Ubc13 and may act as an oxyanion hole^1,12,15^ or to help preorganize the substrates.^14,20–22^ As seen in Figure 4B, mutating the bases has a small effect. The D119A mutation reduces the p*K*_a_ by 0.6 units, the E64A reduces it by 0.3 units and the N79A mutation increases the p*K*_a_ by 0.5 units. These are all modest changes. Furthermore, the D119A and E64A mutants make it more likely that the substrate lysine is deprotonated. This makes sense from a purely electrostatic understanding; a positively charged lysine is stabilized by a negatively charged aspartate or glutamate. The N79A mutant makes it less likely that K63 deprotonates. (The p*K*_a_ of the control residue remains unaffected and is 10.1 ± 0.2.) Although, these small p*K*_a_ shifts are likely within the error bars of the method itself,^31,41^ these data indicate that the D119 and E64 are likely not bases that deprotonate the substrate lysine. Intriguingly, these data also suggest that changes to the structure of the active site caused by the N79A mutation, has a negative effect on the enzyme’s activity. We probed this idea further as seen in Figure 4C.

In Figure 4C, we replicated the WT experiments of Figure 4A with the N79A mutants, i.e., we generated titration curves of K63 in the mutant enzyme in the 0–3, 3.5 Å windows, and an unrestrained simulation. The p*K*_a_ of K63 in the unrestrained window was 9.8 in both the mutant and WT enzymes. In both the 0–3 and 3.5 Å windows, the p*K*_a_ was higher by 0.5 units in the mutant. The control, K94, was 10.1 ± 0.04. These results provide more evidence that disruptions to the hydrogen bond network in the active site have unexpected effects. However, the change in the p*K*_a_ cannot account for the entire rate difference between the WT and N79A mutant. The rate in the N79A mutant is 100–1000× slower than the WT,^20^ whereas an increase of p*K*_a_ by 0.5 would only result in a 3-fold rate reduction.^17,42^

Finally, we examined the hydration of the substrate lysine in the WT enzyme under the assumption that the neutral species will be more stable in a water-free environment. For each distance window, we averaged the number of water molecules within 3 Å of N_ε_ on K63. The results are shown in Table 1. As the lysine enters the active site, it loses one water of hydration: 2.6 ± 0.1 waters in the 7–9 Å window reduces to 1.4 ± 0.1 in the 3.5 Å window. It is likely that the loss of hydration (i.e., desolvation) plays a significant role in the p*K*_a_ reduction.^28^

**Table 1:** Number of Waters within 3 Å of the Amine Nitrogen on K63 in Each *R*_N_ε_–C_ Window

*R*_N_ε_–C_ Window	0–3 Å	3.5 Å	2–4 Å	3–5 Å	5–7 Å	7–9 Å
# of waters	1.3 ± 0.03^a^	1.4 ± 0.1	1.6 ± 0.06	2.4 ± 0.08	2.5 ± 0.2	2.6 ± 0.1

a The standard deviation calculated across the 4, independent simulations.

## IV Conclusions

Ubiquitination is a key reaction in many regulatory pathways. This work focuses specifically on the crucial first step—nucleophile activation—in Ubc13-catalyzed ubiquitination. Defects in the activation mechanism can also have deleterious downstream effects. In fact, certain mutations in the human E2 enzyme UBE2A prevent lysine deprotonation, which leads to developmental disorders.^26^ Therefore, understanding the catalytic mechanisms in ubiquitination enzymes could lead to the development of therapeutics.

We have used constant pH molecular dynamics to show that the deprotonation of K63 in Ubc13-catalyzed polyubiquitination is highly correlated to its distance from the active site and is not due to the action of a nearby generalized base. In fact, the putative bases remain deprotonated throughout the simulation. We have also calculated the p*K*_a_ of K63 and show that it is suppressed by several p*K*_a_ units as it nears the thioester, whereas a nearby control lysine maintains a constant p*K*_a_. We speculate that the p*K*_a_ suppression may be due to the desolvation effect of the active site—as K63 nears the thioester, water is pushed out, destabilizing the charged state.^4,17,28^ Finally, we show that the mutation of a nearby structural residue,^20–22^ N79 to alanine, can affect the active site such that the p*K*_a_ of K63 increases, increasing the energy to nucleophilic activation.

## Supplementary Material





## Notes

Custom force field parameters for the thioester bond, including partial charges and the improper torsion; backbone RMSDs; and the SI cites refs 14,21,34,35 (PDF)
AMBER input and parameter files for the MD simulations (ZIP)
