# Supplementary material for: The Lysine Deprotonation Mechanism in a Ubiquitin Conjugating Enzyme
Source: J Phys Chem B. 2025 May 12;129(20):4962–8. doi: 10.1021/acs.jpcb.5c01486 (PMC12105026; doi:10.1021/acs.jpcb.5c01486)
Supplement: Supplementary file 1 [file jp5c01486_si_001.pdf]

Supporting Information

The Lysine Deprotonation Mechanism in a Ubiquitin Conjugating Enzyme

Alexis J. Wathan[1], Nicole M. Deschene[2,‡], Joseph M. Litz[2,‡], and Isaiah Sumner[2,*]

[1] Department of Science and Mathematics, Rochester Institute of Technology/NTID, Rochester, NY, 14623, United States

[2] Department of Chemistry and Biochemistry, James Madison University, Harrisonburg, VA, 22807, United States

†Authors made equal contributions

*Correspondence:
I. Sumner
901 Carrier Drive, MSC 4501
Harrisonburg, VA, 22807, United States
Phone: +1-540-568-6670
Email: sumneric@jmu.edu

Thioester force-field parameters

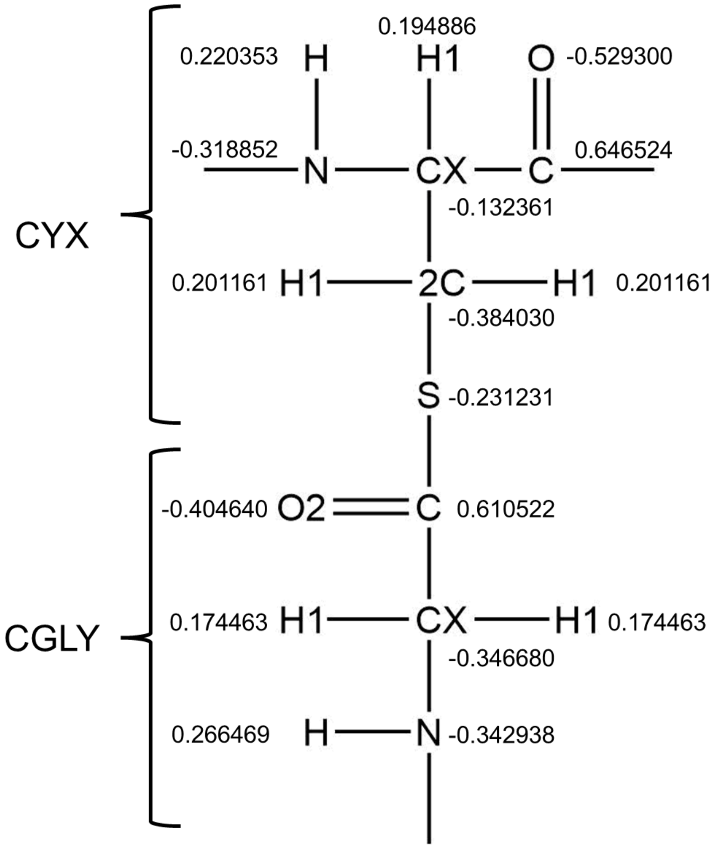

Figure S1 RESP[1] derived charges for the thioester bond.[2] The Amber atom types are listed and force field parameters are taken from the Amber CYX and CGLY residues. Missing parameters were taken from GAFF (see below).[3] An improper torsion term was parameterized (see below)[4] and added to keep the thioester bond (CX-S-C-O2) planar.

Based on GAFF

MASS

BOND

S-C 261.9 1.7620 From gaff c-ss

ANGLE

S-C-CX 78.990 114.320 from gaff c3-c-ss

S-C-O2 81.780 122.290 from gaff o-c-ss

2C-S-C 38.470 100.290 from gaff c-ss-c3

DIHE

2C-S-C-CX 2 6.200 180.000 2.000 from gaff X -c -ss-X

2C-S-C-O2 2 6.200 180.000 2.000 from gaff X -c -ss-X

IMPROPER

CX-S-C-O2 15.3 180.000 2.0 from fitting to m062x/def2tzvp

NONBON

CpHMD Restraint Data and Backbone RMSDs

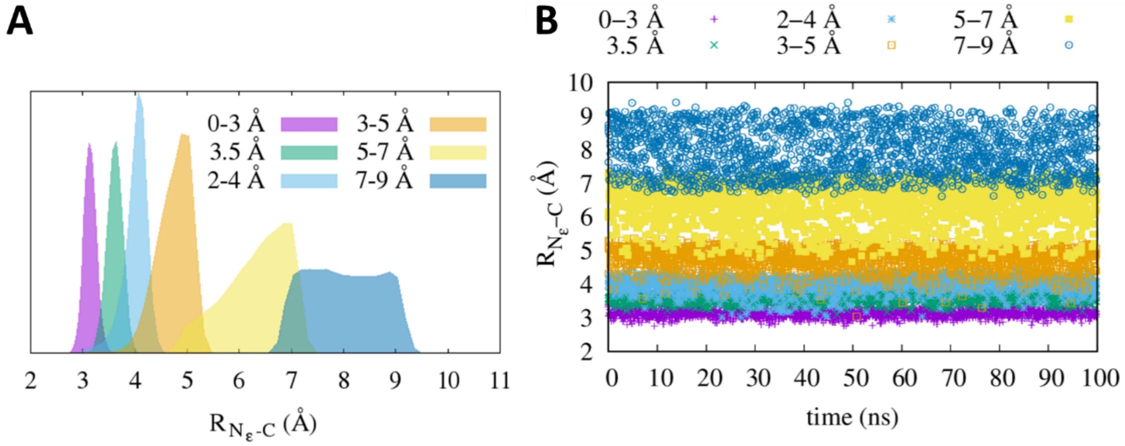

Figure S2 (A) Histograms of the K63/thioester distance $R_{N\varepsilon\text{-}C}$ and (B) $R_{N\varepsilon\text{-}C}$ vs simulation time show that the entire distance range is well covered (A). The different distance windows are indicated by different color histograms (A) or by different color and shapes of points (B).

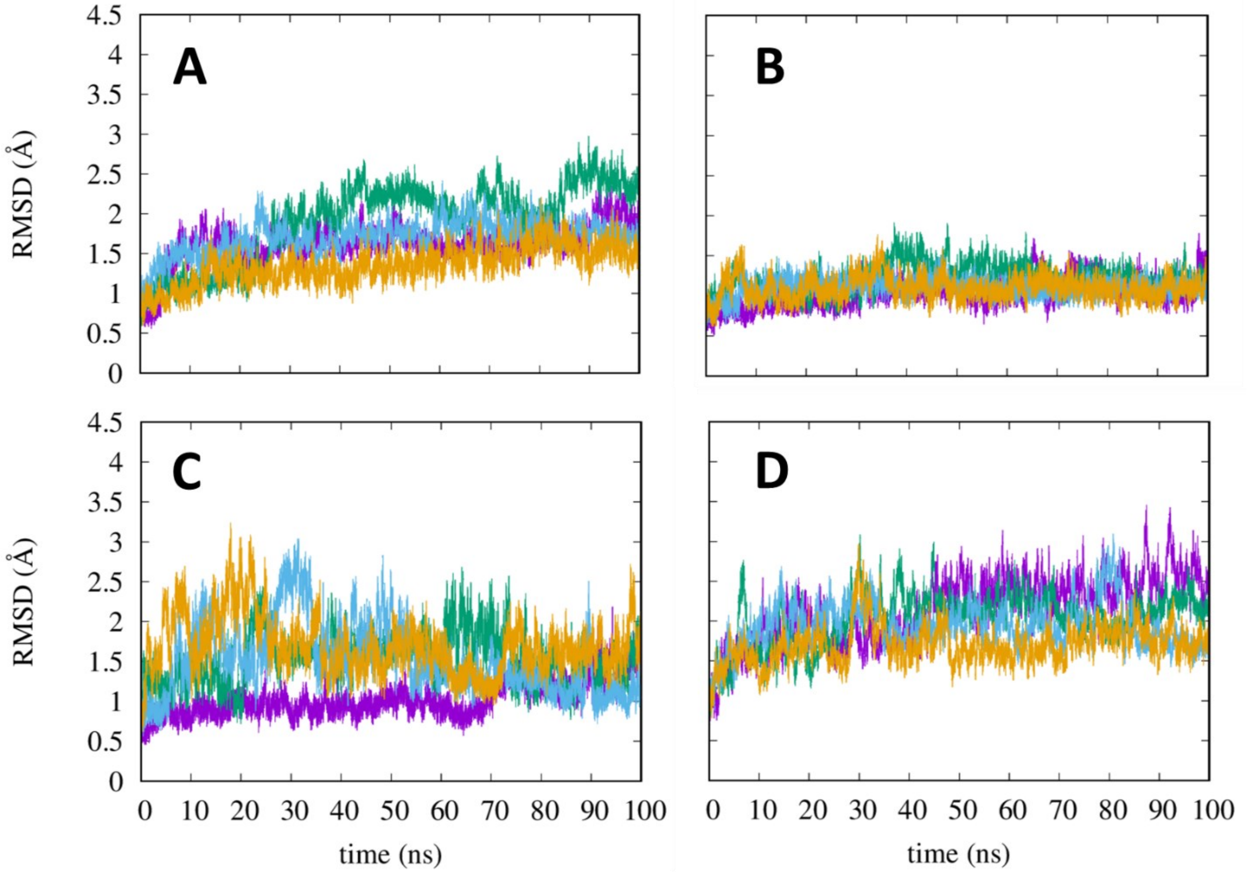

Figure S3 Backbone RMSDs from the 0-3 Å window CpHMD simulations for (A) the Ubc13~Ub conjugate, (B) the UeV ,(C) Ub* , and (D) the entire protein system . Each independent simulation is shown in a different color.

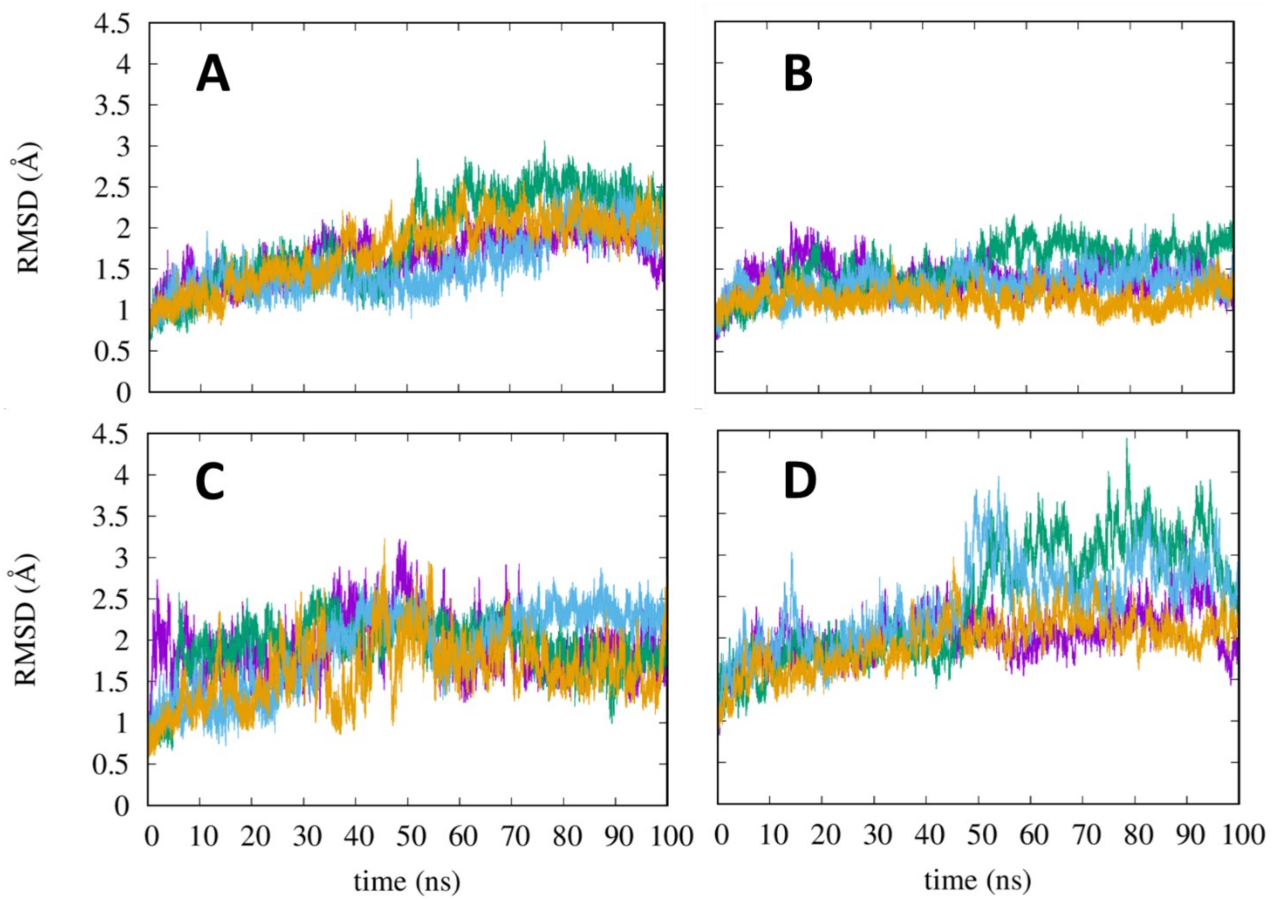

Figure S4 Backbone RMSDs from the 3.5 Å window CpHMD simulations for (A) the Ubc13~Ub conjugate, (B) the UeV, (C) Ub*, and (D) the entire protein system. Each independent simulation is shown in a different color.

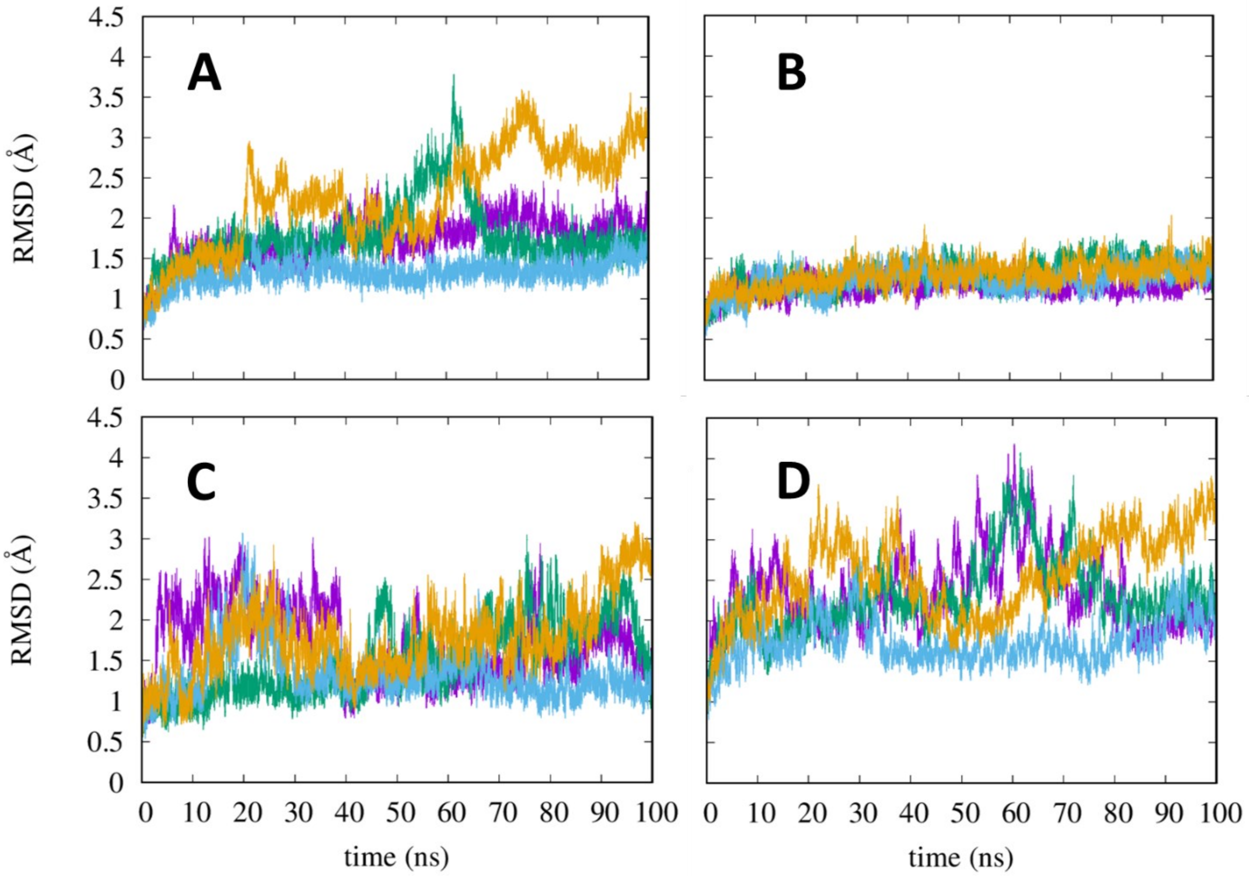

Figure S5 Backbone RMSDs from the 2-4 Å window CpHMD simulations for (A) the Ubc13~Ub conjugate, (B) the UeV, (C) Ub*, and (D) the entire protein system. Each independent simulation is shown in a different color.

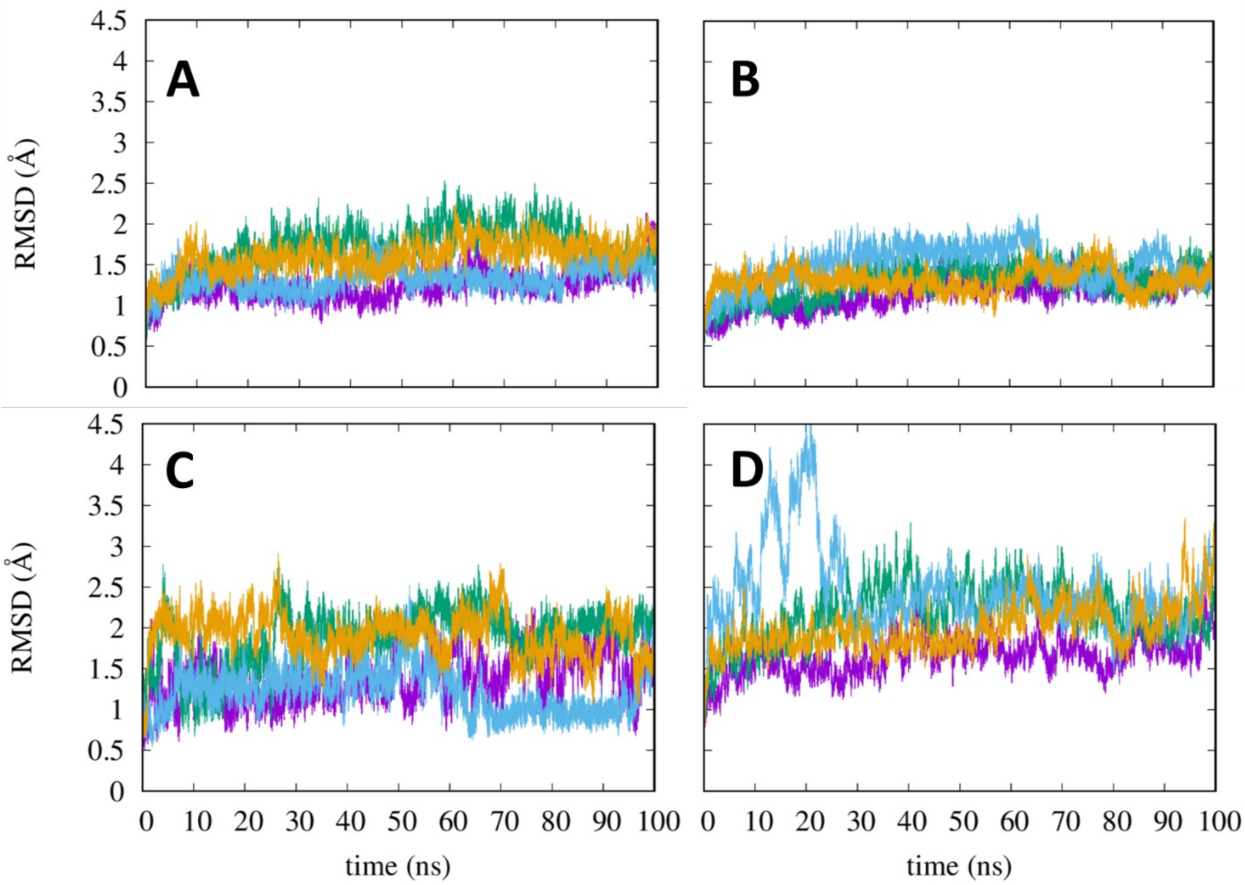

Figure S6 Backbone RMSDs from the 3-5 Å window CpHMD simulations for the (A) Ubc13~Ub conjugate, (B) the UeV, (C) Ub*, and (D) the entire protein system. Each independent simulation is shown in a different color.

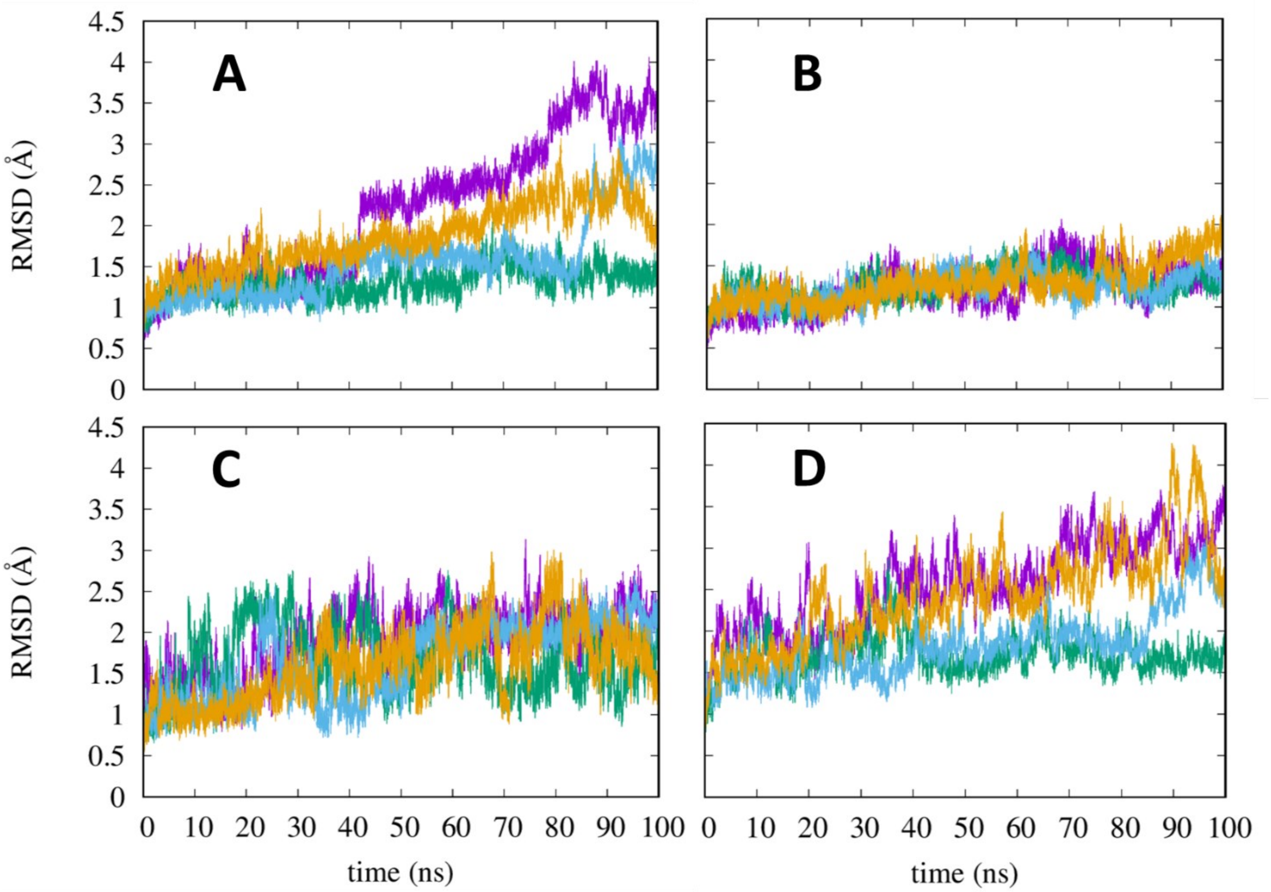

Figure S7 Backbone RMSDs from the 5-7 Å window CpHMD simulations for (A) the Ubc13~Ub conjugate, (B) the UeV, (C) Ub*, and (D) the entire protein system. Each independent simulation is shown in a different color.

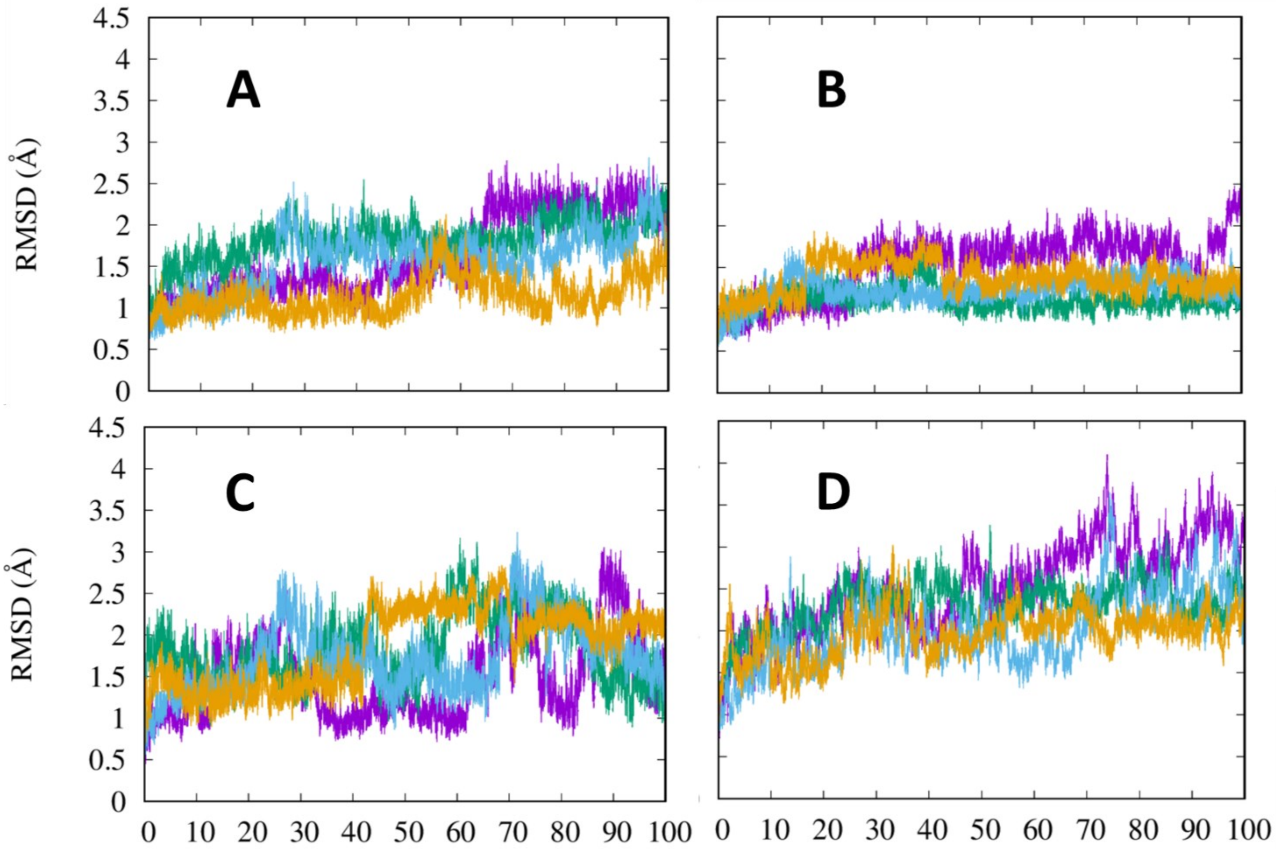

Figure S8 Backbone RMSDs from the 7-9 Å window CpHMD simulations for (A) the Ubc13~Ub conjugate, (B) the UeV, (C) Ub*, and (D) the entire protein system. Each independent simulation is shown in a different color.

pH-REMD Restraint Data

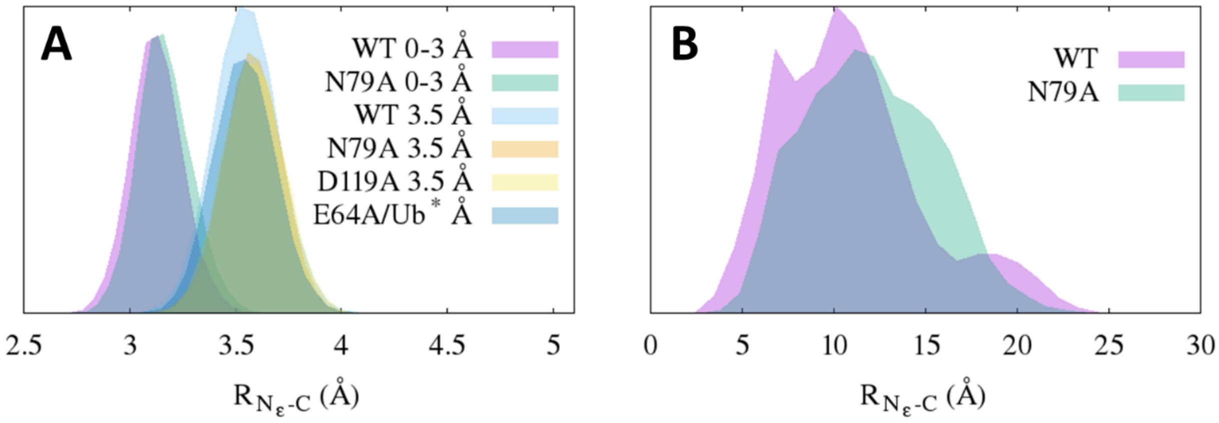

Figure S9 Histograms of the K63/thioester distance $R_{N\varepsilon\text{-}C}$ for (A) restrained and (B) unrestrained pH-REMD simulations. The different distance windows and WT/mutants are indicated by different color histograms.

References

(1) Bayly, C. I.; Cieplak, P.; Cornell, W.; Kollman, P. A. A Well-Behaved Electrostatic Potential Based Method Using Charge Restraints for Deriving Atomic Charges: The RESP Model. *The Journal of Physical Chemistry* **1993**, *97* (40), 10269–10280. https://doi.org/10.1021/j100142a004.

(2) Wilson, R. H.; Zamfir, S.; Sumner, I. Molecular Dynamics Simulations Reveal a New Role for a Conserved Active Site Asparagine in a Ubiquitin-Conjugating Enzyme. *Journal of Molecular Graphics and Modelling* **2017**, *76*, 403–411. https://doi.org/10.1016/j.jmgm.2017.07.006.

(3) Wang, J.; Wolf, R. M.; Caldwell, J. W.; Kollman, P. A.; Case, D. A. Development and Testing of a General Amber Force Field. *Journal of Computational Chemistry* **2004**, *25* (9), 1157–1174. https://doi.org/10.1002/jcc.20035.

(4) Johnson, J.-A. K.; Sumner, I. On the Possibility That Bond Strain Is the Mechanism of RING E3 Activation in the E2-Catalyzed Ubiquitination Reaction. *Journal of Chemical Information and Modeling.* **2022**, *62* (24), 6475–6481. https://doi.org/10.1021/acs.jcim.2c00423.
